# Model proposal for calculating waste associated with processing consigned surgical instruments *

**DOI:** 10.1590/1518-8345.6716.4062

**Published:** 2023-12-04

**Authors:** Simone Garcia Lopes, Vanessa de Brito Poveda

**Affiliations:** 1 Universidade de São Paulo, Escola de Enfermagem, São Paulo, SP, Brasil.; 2 Centro Universitário Faculdade de Medicina do ABC, Faculdade de Enfermagem, Santo André, SP, Brasil.

**Keywords:** Perioperatory Nursing, Costs and Cost Analysis, Elective Surgical Procedures, Health Assessment, Hospital Costs, Sterilization, Enfermería Perioperatoria, Costos y Análisis de Costos, Procedimientos Quirúrgicos Electivos, Valoración de Salud, Costos de Hospital, Esterilización, Enfermagem Perioperatória, Custos e Análise de Custos, Procedimentos Cirúrgicos Eletivos, Avaliação em Saúde, Custos Hospitalares, Esterilização

## Abstract

**Objective:**

to evaluate the waste generated from processing surgical instruments consigned in elective orthopedic surgeries and propose a model for calculating waste associated with processing consigned surgical instruments.

**Method:**

a quantitative, descriptive-exploratory case study carried out in a large university hospital in two phases: (1) retrospective by consulting administrative records of canceled elective orthopedic surgeries, with provision for the use of consigned materials for identification of the sub-specializations with the greatest demand; and (2) prospective through direct, non-participant observations of processing consigned surgical instruments prepared for the identified surgeries and proposition of a model for calculating waste associated with processing these materials.

**Results:**

hip arthroplasty, spine arthrodesis and knee arthroplasty surgeries were identified as presenting the greatest demand, resulting in 854 boxes of consigned surgical instruments processed and unused. Processing waste was estimated at R$34,340.18 (US$6,359.30).

**Conclusion:**

the proposed equation made it possible to calculate the waste related to the production and non-use of boxes of surgical instruments consigned for orthopedic procedures and can equip nurses for planning based on institutional, care and financial data, aiming to make better use of resources through waste identification.

## Abstract (es)

**Objetivo:**

evaluar el desperdicio generado por el procesamiento de instrumental quirúrgico consignado en cirugías ortopédicas electivas y proponer un modelo para el cálculo del desperdicio asociado al procesamiento de instrumental quirúrgico consignado.

**Método:**

estudio de caso, cuantitativo, descriptivo-exploratorio, realizado en un hospital universitario grande, en dos fases: (1) retrospectiva, mediante la consulta de registros administrativos de cirugías ortopédicas electivas canceladas, con previsión de uso de materiales consignados, para identificar las subespecialidades con mayor demanda; y (2) prospectiva, por medio de observaciones directas, no participantes, del procesamiento de instrumental quirúrgico consignado preparado para las cirugías identificadas, y de la propuesta de un modelo para el cálculo del desperdicio asociado al procesamiento de estos materiales.

**Resultados:**

se identificaron las cirugías de artroplastia de cadera, artrodesis de columna y artroplastia de rodilla con mayor demanda, resultando en 854 cajas de instrumental quirúrgico consignado procesado y sin uso. El desperdicio del procesamiento se estimó en R$ 34.340,18 (US$ 6,359.30).

**Conclusión:**

la ecuación propuesta permitió calcular el desperdicio relacionado con la producción y la no utilización de cajas de instrumental quirúrgico consignado para procedimientos ortopédicos, y puede equipar a los enfermeros para la planificación basada en datos institucionales, asistenciales y financieros, con el objetivo de aprovechar mejor los recursos por medio de la identificación del desperdicio.

## Abstract (pt)

**Objetivo:**

avaliar o desperdício gerado pelo processamento de instrumentais cirúrgicos consignados em cirurgias ortopédicas eletivas e propor um modelo de cálculo de desperdício associado ao processamento de instrumentais cirúrgicos consignados.

**Método:**

estudo quantitativo, descritivo-exploratório, do tipo estudo de caso, realizado em hospital universitário de grande porte, em duas fases: (1) retrospectiva pela consulta aos registros administrativos de cirurgias ortopédicas eletivas canceladas, com previsão de uso de materiais consignados para identificação das subespecialidades com maior demanda e (2) prospectiva por meio de observações diretas, não participantes, do processamento de instrumentais cirúrgicos consignados preparados para as cirurgias identificadas e proposição de modelo de cálculo de desperdício associado ao processamento desses materiais.

**Resultados:**

foram identificadas as cirurgias de artroplastia de quadril, artrodese de coluna e artroplastia de joelho, com maior demanda, resultando em 854 caixas de instrumentais cirúrgicos consignados processados e não utilizados. O desperdício do processamento foi estimado em R$ 34.340,18 (US$ 6,359.30).

**Conclusão:**

a equação proposta permitiu calcular o desperdício relacionado à produção e não utilização de caixas de instrumentais cirúrgicos consignados para procedimentos ortopédicos e pode instrumentalizar os enfermeiros para o planejamento pautado em dados institucionais, assistenciais e financeiros, visando ao melhor aproveitamento dos recursos através da identificação do desperdício.

**Highlights:**

**(1)** Calculation of waste associated with processing surgical instruments.

**(2)** Processing consigned materials.

**(3)** Costs related to consigned materials.

**(4)** Proposal for a waste calculation model.

## Introduction

International scientific literature reveals that surgical procedures account for 40% of a hospital’s total expenses due to the large mobilization of human, material and technological resources ^(^ 1 ^-^ 2 ^)^. Thus, the surgical cancellation rate is one of the indicators used to evaluate the efficiency of a service and considers any reason that led to this outcome, whether related to the patient or the hospital institution ^(^ 2 ^-^ 4 ^)^. 

National and international evidence reveals that orthopedic surgeries are among the surgical specializations with the highest cancellation rate ^(^ 2 ^,^ 4 ^)^. It is further worth noting that the cancellation of a surgical procedure causes financial impacts for the hospital, as materials and professional work are wasted by various professionals and sectors, such as the Pharmaceutical, Material and Sterilization Center and the Surgical Center ^(^ 1 ^,^ 3 ^)^ , delay in meeting qualitative and quantitative production targets, idleness in the operating room, in addition to negative aspects for the health and quality of life of patients, generating physical and psychological exhaustion. 

Brazilian legislation establishes that the Material and Sterilization Center (MSC) is responsible for processing health products (HPs) that will be used in any procedure which takes place in the hospital in its entirety, meaning from their cleaning to disinfection and/or sterilization, regardless of whether it is owned by the hospital, privately owned by the medical team or consigned from companies supplying materials ^(^ 5 ^-^ 6 ^)^ , with the nursing team in Brazil being responsible for all work carried out at the MSC. 

Consigned materials are among those which do not belong to the hospital and are used very frequently. The consignment of materials represents a loan by the supplier company to the hospital of boxes of non-sterile surgical instruments of different types and sizes, of implantable materials, and a single procedure may use several boxes. Boxes of consigned surgical instruments are delivered a few hours before the surgical procedure, which are then processed by the MSC and returned to the supplying company as soon as they are used, or even unused in cases of surgical cancellation.

The management of consigned materials constitutes a challenge for professionals working in MSCs worldwide, leading hospitals to seek proposals to improve and standardize protocols ^(^ 6 ^-^ 8 ^)^. The Manual of Good Management Practices for Orthoses, Prostheses and Special Materials ( *Manual de Boas Práticas de Gestão das* Órteses *, Próteses e Materiais Especiais - OPME*) in Brazil offers guidelines for standardizing *OPME* acquisition, request, receipt, storage, dispensing, use and control activities ^(^ 9 ^)^. 

Depending on the characteristics of the consigned materials the MSC processes, it is essential that each one makes its own time estimate for receiving and preparing the material, considering that time is a finite and valuable resource, and knowing how to use it appropriately can reduce stress and increase productivity ^(^ 10 ^)^. 

The nursing team participates in all stages involving HP processing, especially consigned ones ^(^ 5 ^,^ 11 ^)^. It is worth noting that the return of processed and unused temporary consigned materials as a result of surgical cancellation, for any reason, highlights wasted production and a cost which needs to be estimated by health institutions. 

Rising healthcare costs require reduced waste and greater hospital efficiency. Financial decisions and budgetary planning of health institutions increasingly require the participation of nurses as direct managers of human and material resources ^(^ 7 ^,^ 12 ^-^ 13 ^)^. 

MSC nurses have an unquestionable participation in HP processing due to their technical responsibility and academic training. With advances in technology in the sector and the production of new scientific evidence, nurses have also had to develop administrative and management skills. From this perspective, nurses take control of resources, thereby mitigating waste opportunities.

The process of preparing consignment products deserves a microeconomic analysis of all its stages, identifying the dedicated materials and human resources, highlighting the appropriate number of professionals and the preparation time. This analysis can summarize the processes, their costs and direct actions to optimize financial resources.

The MSC is often identified by hospital administrators as a unit which generates high costs; thus, there is a need to demonstrate the values invested in each stage of the process through management tools for more efficient management and to provide a balance between what is used and what is necessary, establishing standards and enabling an analysis of losses in cancelled surgery cases ^(^ 12 ^)^. 

Therefore, the aim herein is to offer perioperative nurses a support model which enables them to understand their own reality in depth and contributes to implementing management indicators which help to improve the management of financial resources. In view of the above, the following question arises: what is the cost of processing consigned and unused orthopedic surgical instruments?

Consequently, this study aimed to: 1) evaluate the waste generated by processing temporary consigned surgical instruments, prepared and not used in elective orthopedic surgeries which have greater demand for consigned boxes; 2) calculate the mean direct cost (MDC) of the processing steps for boxes of consigned orthopedic surgical instruments; 3) estimate the total cost (TC) of surgical cancellation related to processing boxes of prepared and unused consigned orthopedic surgical instruments; and 4) propose a model for calculating waste associated with processing consigned surgical instruments.

## Method

### Study design

This is a descriptive-exploratory case study (CS). CSs investigate a contemporary phenomenon in depth, and in its real-world context, especially when the boundaries between the phenomenon and the context are not clearly evident. CSs enable evaluating multiple variables, and can draw on multiple evidence sources (interviews, documents, artifacts and direct observation of the event) ^(^ 14 ^)^. 

### Scenario

The study was conducted in a large, highly complex university hospital with 316 beds, located in the city of Santo André, SP, Brazil. It has a management contract with the State of São Paulo as a Social Health Organization ( *Organização Social de Saúde - OSS*) to offer health services to the population and manage financial resources, offering a teaching field for a university with undergraduate and postgraduate courses in the health field (medicine, nursing, physiotherapy, nutrition, occupational therapy, and psychology, among others). 

The hospital has a Surgical Center with 11 operating rooms, and performs an average of 800 surgical procedures/month across all specializations. Orthopedic surgeries use consigned surgical instruments, and are responsible for around 12% of scheduled surgeries. The MSC is centralized, operates 24 hours a day, and processes an average of 8,000 critical health products per month.

The MSC of the study scenario currently has the following equipment: two thermo-disinfector washers, two benchtop ultrasonic washers, three steam sterilizers (autoclaves) and a low temperature sterilizer (hydrogen peroxide plasma).

The MSC team of nursing professionals is composed of six nurses (NUs) and 28 nursing assistants (NAs). Due to the high number of surgeries which use *OPME* (an average of 95 surgeries per month), the MSC has a daytime NU and a nighttime NU dedicated only to this demand. 

The MSC of the study hospital has a consignment contract which establishes the permanent loan of consigned HPs, which means the materials are provided by the consignment company and invoiced in case of use; therefore, they remain in its own stock, and the temporary loan in case of materials which are used less frequently or have a high acquisition cost must be delivered by the consignment company and returned following their use or not ^(^ 9 ^)^. 

Therefore, the temporary consigned materials (which are the object of this study) are provided by external companies, with the MSC nursing team being responsible for receiving this material; this involves checking the ordered materials, their inspection, cleaning and sterilization. As it is consigned material, meaning it does not belong to the hospital, all consigned materials not used in a surgery due to surgical cancellation, whether or not they have been placed on the operating table, will be unpacked and returned clean to the consignment company ^(^ 9 ^)^. Thus, if the procedure is rescheduled, the supply, cleaning and sterilization process will be carried out again, characterizing waste. 

### Period

The investigation was carried out in two phases: in phase 1 (retrospective), surgeries canceled in 2019, 2020 and 2021 were included, with data collected between March and May 2022; in phase 2 (prospective), direct and non-participant observations were performed in the period between June and October 2022.

### Sample and selection criteria

The sample was composed by convenience in both phases of the investigation, namely: in phase 1, administrative records of canceled elective orthopedic surgeries of subjects over 18 years of age, with provision for the use of temporary implantable/consigned materials, but which were canceled after the delivery and preparation of these instruments at the MSC in the years 2019, 2020 and 2021 were consulted. Incomplete records were excluded; then in phase 2, the processing stages of boxes of consigned orthopedic surgical instruments which presented a greater demand (hip arthroplasty, spine arthrodesis and knee arthroplasty) were monitored; thus, five direct, non-participant observations of processing consigned surgical instruments allocated in surgical boxes for hip arthroplasty, spine arthrodesis and knee arthroplasty procedures were performed, totaling 15 observations. To this end, three nurses and eight nursing assistants, over 18 years of age, all with more than six months of experience in the MSC, who processed consigned surgical instruments, and were under contract of 144 hours per month (36 hours/ weekly) were observed. Any observation that did not comply with any mandatory step in processing orthopedic surgeries was excluded.

### Data collection

Data collection took place between March and October 2022 in two phases, following the recommended steps for preparing a case study ^(^ 14 ^)^. 

In Phase 1, retrospective data collection was conducted by the researcher herself from administrative files (accounting reports, medical records and maps of scheduled and canceled elective orthopedic surgeries) from the years 2019, 2020 and 2021, which included forecasts for the use of consigned material. Thus, a data collection instrument was used (developed by the researchers) containing information regarding the procedure and materials (type of surgery, reason for cancellation, number of boxes of consigned surgical instruments prepared and not used).

In Phase 2, three previously trained nursing professionals monitored the nursing team in all processing stages of boxes of consigned surgical instruments used in hip arthroplasty, spine arthrodesis and knee arthroplasty surgeries through observation and measuring the time spent (timed).

The focus of non-participatory observation in Phase 2 was to identify the professional category (nursing assistant and nurse) dedicated to the function evaluated, the number of professionals dedicated to the different stages of processing, inputs used and the time dedicated to each stage (S1 - Receiving and Verification; S2 - Evaluation and Manual Cleaning; S3 - Automated Cleaning; S4 - Inspection and Preparation; S5 - Sterilization and Storage; and S6 - Sterilization Tests).

### Data analysis and treatment

The collected data were organized in electronic databases and presented in absolute numbers, mean, standard deviation (SD), medians, minimum and maximum values, absolute and relative frequency.

The coefficient of variation (CV) was used to present the variation in material costs, since CV is used in statistics to compare the variation of a set of data that may differ from the mean. The R version 4.2.2 program was used for statistical analysis. The values to calculate costs were expressed in Reais (R$), and considered the reference value of US$5.40 = R$1.00, considering the average exchange rate in 2021.

### Ethical aspects

The study was approved by the Ethics Committee under number 5,243,445. The study participants received guidance on how to perform the research and expressed their agreement to participate in the investigation by signing the Informed Consent Form. The study followed the recommendations of the Revised Standards for Quality Improvement Reporting Excellence (SQUIRE 2.0) ^(^ 15 ^)^. 

The present study was conducted with support from the *Coordenação de Aperfeiçoamento de Pessoal de Nível Superior* - *Brasil* ( *CAPES*) - Financing code 001. 

## Results

In the evaluated period between 2019 and 2021, there was waste associated to the processing of 1,640 boxes of temporary consigned surgical instruments related to 353 (100%) canceled surgeries, being 157 (44.5%) in 2019; 113 (32.0%) in 2020; and 83 (23.5%) in 2021. The sub-specializations of hip arthroplasty, spine arthrodesis and knee arthroplasty surgeries suffered 85 (24.1%) cancellations, accounting for 854 (52%) boxes of consigned surgical instruments produced and not used. The other orthopedic sub-specializations analyzed were distributed into 48% of canceled procedures, included pediatric, sports, hand, shoulder, oncology and foot procedures.

Causes related to the institution stood out (40; 47.0%) among the reasons for cancellation of evaluated hip, spine and knee surgeries between 2019 and 2021, highlighting problems with the material, including inadequate material for use, such as wet material, presence of dirt and delay in preparation; lack of material or incorrect material for the procedure, which included the delivery of wrong or incomplete instruments; next, patient-related problems (39; 45.9%), especially clinical ones; and finally, problems related to the medical team (6; 7%).

The analysis revealed that the night period was responsible for 55 (73.3%) of the processing of consigned hip, knee and spine surgical instruments, thus, as a reference for calculating the cost of labor value/minute, it was night salary was used ( Table 1). 

It is noteworthy that “Stage 3 - Automated cleaning” is not presented in Tables 1and 2, since it is a fully automated process, meaning it does not involve labor costs, and its loading was analyzed in Stage 2 (manual cleaning). 

**Table 1 -:** Number of processing observations of consigned boxes per period (n*=15). Santo André, SP, Brazil, 2022

Period	S1 ^†^	S2 ^‡^	S4 ^§^	S5 ^||^	S6 ^¶^	Total
Morning (n ^*^; % ^**^)	1 (6.7)	0	1 (6.7)	1 (6.7)	0	3 (4)
Afternoon (n ^*^; % ^**^)	7 (46.7)	5 (33.3)	2 (13.3)	1 (6.7)	2 (13.3)	17 (26.7)
Night (n ^*^; % ^**^)	7 (46.7)	10 (66.7)	12 (80)	13 (86.7)	13 (86.7)	55 (73.3)
Total (n ^*^; % ^**^)	15 (100)	15 (100)	15 (100)	15 (100)	15 (100)	75 (100)

^*^n = Absolute number; ^†^S1 = Receiving and Verification; ^‡^S2 = Evaluation and Manual Cleaning; ^§^S4 = Inspection and Preparation; ^||^S5 = Sterilization and Storage; ^¶^S6 = Sterilization Tests; ^**^% = Percentage

Five observations were performed per type of surgery (hip, spine and knee), meaning five surgeries of each type, totaling 15 complete processes of consigned surgical instruments. It was observed that an average of 12 boxes of surgical instruments were prepared for hip surgery, seven for spine surgery and 11 for knee surgery. The average estimate of boxes of these materials by type of surgery was considered to calculate the average processing cost.

In aiming to maintain the safety and quality of processing and after careful analysis of the number of pieces present in each box, the complexity, the weight and dimensions of the boxes, the nursing professional in charge of processing then assessed the need and distributed the instruments surgical items consigned in new boxes, increasing the number of boxes processed compared to those received.

Only the labor cost of assistants and nurses was considered with regard to the professional category, since there were no nursing technicians on the MSC team. The night salary was the reference for calculating labor costs per minute, as this was the shift that invested the most time in processing boxes of consigned surgical instruments. The monthly salary of daytime nurses is R$8,351.22 (US$1,546.52) and for nighttime nurses it is R$9,907.29 (US$1,834.68). The nursing assistants receive R$4,638.54 (US$859.00) for daytime work and R$5,410.42 (US$1,002.00) for nighttime work.

It appears that Step 4 (inspection and preparation) consumed most of the MSC professional’s time in preparing consigned surgical instruments, and is therefore responsible for higher costs. The time in minutes recorded in preparing surgical instruments allocated for surgeries is presented in Table 2. 

Table 3 shows the average value paid and the percentage of materials used at each stage in preparing surgical instruments assigned for hip, knee and spine surgeries, and the labor cost and its percentage for each preparation stage. 

Automated cleaning (Step 3) does not involve labor time, as the time to supply the machinery was considered in Step 2 (manual cleaning). The labor dedicated to storage was considered to calculate Stage 5 (sterilization and storage), as the cost of the sterilization process (parts and accessories, maintenance material, water and sewage and electricity) was weighted in the indirect cost.

The MSC unit has a computerized financial report which presents the average unit cost of processed articles, produced through the sum of all direct, indirect and apportioned costs (total cost) and divided by the total number of articles produced in a period.

**Table 2 -:** Time and labor cost per stage of processing boxes of surgical instruments consigned for hip (n*=12 boxes), spine (n*=07 boxes) and knee (n*=11 boxes) surgeries. Santo André, SP, Brazil, 2022.

Sub-specialization	Professional category	S1 ^†^ (min**)	S2 ^‡^ (min**)	S4 ^§^ (min**)	S5 ^||^ (min**)	S6 ^¶^ (min**)	Total (min**)	Value/min** (R$ ^‡‡^)	Labor cost (R$ ^‡‡^)	Labor cost box R$ ^‡‡^ (US$ ^§§^)
Hip	Nurse	0	0	64.8	4	6.8	75.6	1.15	86.94	
	Nurse assistant	32.8	40	38.2	19.8	12	142.8	0.63	89.96	
	Hip total	32.8	40	103	23.8	18.8	218.4	1.78	176.9	14.74 (2.73)
Spine	Nurse	0	0	32.4	4	4.4	40.8	1.15	46.92	
	Nurse assistant	29.2	27	34	13.4	11.2	114.8	0.63	72.32	
	Spine total	29.2	27	66.4	17.4	15.6	155.6	1.78	119.2	17.03 (3.15)
Knee	Nurse	0	0	54.4	4	4.4	62.8	1.15	72.22	
	Nurse assistant	35.2	70	51.4	12	12	180.6	0.63	113.78	
	Knee total	35.2	70	105.8	16	16.4	243.4	1.78	186.00	16.90 (3.13)

Considering 1 dollar = R$5.40, mean exchange rate in 2021; ^*^n = Absolute number; ^†^S1 = Receiving and Verification; ^‡^S2 = Evaluation and Manual Cleaning; ^§^S4 = Inspection and Preparation; ^||^S5 = Sterilization and Storage; ^¶^S6 = Sterilization tests; ^**^min = minute; ^‡‡^R$ = Reals; ^§§^US$ = Dollar

**Table 3 -:** Cost of materials and labor used in processing consigned boxes for hip (n*=12), spine (n*=7) and knee (n*=11) surgeries. Santo André, SP, Brazil, 2022.

Inputs	Value of materials R$ ^†^ (US$ ^‡^)			Value of materials percentage ^(§)^			Labor value R$ ^†^ (US$ ^‡^)			Labor value percentage ^(§)^			Total cost R$ ^†^ (US$ ^‡^)		
	Hip	Spine	Knee	Hip	Spine	Knee	Hip	Spine	Knee	Hip	Spine	Knee	Hip	Spine	Knee
S1 ^||^															
Nitrile procedure gloves	1.22	1.22	1.22	5.60	6.22	5.22	20.64	18.39	22.17	94.40	95.48	94.78	21.86	19.61	23.39
S2¶															
Nitrile procedure gloves	3.66	3.66	3.66	22.42	29.97	15.78	25.20	17.01	44.20	77.58	70.03	84.22	32.48	24.29	52.48
Enzymatic Detergent	0.80	0.80	0.80												
Surgical compress	2.82	2.82	2.82												
S3 ^**^															
Enzymatic Detergent	5.76	2.88	5.76	100	100	100	0.0	0.0	0.0	0.0	0.0	0.0	5.76	2.88	5.76
S4 ^††^															
Chemical Integrator	4.68	2.73	4.29	61.25	61.17	36.82	98.58	58.68	94.94	38.75	38.83	63.18	254.38	151.15	150.26
Disposable towel	4.56	2.66	4.18												
Masking Tape (m ^‡‡^)	12.00	7.00	11.00												
Autoclave Tape	0.24	0.28	0.44												
NW/SMS ^§§^	8.44	0.0	0.0												
NW/SMS ^§§^	125.4	79.8	125.4												
S5/S6 ^||||^															
Bowie Dick Test	0.38	0.38	0.38	51.38	57.68	58.32	32.43	25.16	24.78	48.62	42.32	41.68	66.71	59.44	59.44
Biological Test	33.9	33.9	33.9												
Total	203.86	138.13	193.85				176.85	119.24	186.09				380.71	257.37	379.94
	(37.75)	(25.58)	(35.90)				(32.75)	(22.08)	(34.46)				(70.50)	(47.66)	(70.36)

For calculation, two enzymatic detergent cycles were considered in the automated cleaning process (72 ml); To calculate the workforce, the salary for nurses is R$9,907.29 and R$5,410.42 for nursing assistants; Considered 1 dollar = R$ 5.40, mean exchange rate in 2021; ^*^n = Absolute Number; ^†^R$ = Reals; ^‡^US$ = Dollar; ^§^% = Percentage; ^||^S1 = Receiving and Verification; ^¶^S2 = Evaluation and Manual Cleaning; ^**^S3 = Automated Cleaning; ^††^S4 = Inspection and Preparation; M ^‡‡^ = Meters; ^§§^NW/SMS = Non-woven/Spundbond-Meltblow-Soundbond; ^||||^S5/S6 = Sterilization and Storage, Sterilization Tests

The absorption costing method is used by the institution under analysis in the present study, and presents the total cost of the MSC and disregards the complexity, the rental of materials and labor, and the steps that involve the processing to create the unit cost of the articles, making it impossible to identify the cost of automated cleaning and sterilization.

The management reports in 2021 presented an average cost of R$19.11 (US$3.54) per object processed, regardless of the type of article, complexity and/or processing to which it was subjected. In view of these facts, and seeking to offer the TC for processing boxes of surgical instruments consigned for hip, spine and knee surgeries using the total cost of the MSC, the redistribution of indirect costs, expenses and apportionments was conducted according to the volume processed and sterilized. Then, the costs were distributed according to management reports obtained at the study institution, and are presented as direct, fixed and variable costs.

The indirect cost value (IC) of R$6.10 (US$1.13) was identified, added to the direct cost (DC) of the boxes of consigned surgical instruments processed for hip, spine and knee surgeries to obtain the Total Cost (TC) per box processed.

Thus, considering the 85 cases of cancellations in the analyzed period from 2019 to 2021 only for hip, spine and knee orthopedic procedures, they generated an estimated processing of 854 boxes. In analyzing the financial waste associated with preparing consigned surgical instruments in the evaluated period, the institution incurred costs for canceling hip surgery equal to R$11,610.74 (US$2,150.14); spine surgery cancellations resulted in losses of R$12,343.68 (US$2,285.89); and knee procedure cancellations resulted in losses of R$10,525.76 (US$1,949.21), totaling R$34,340.18 (US$6,359.30).

In view of the results presented, and with the purpose of providing instruments to the MSC nurse manager and other professionals dedicated to knowing and controlling the possible waste of a health institution, equation 1 presents the proposed model to estimate the Expected Waste (EW) resulting from processing prepared and unused boxes of consigned surgical instrument, due to the cancellation of surgeries. Equation 2 makes it possible to calculate the DC by type of surgery.

*Equation 1*

\begin{equation*}EW = \left(IC \sum_{i=1}^bn_i+CD\right)P\ (cancelled\ surgery)\end{equation*}

Legend: \begin{equation*}EW\end{equation*} = *expected waste*

\begin{equation*}IC\end{equation*} = *indirect cost*

\begin{equation*}n_i\end{equation*} = *(n) number of boxes (i) types of boxes*

\begin{equation*}\sum_{i=1}^bn_i\end{equation*} = *sum of the types of boxes*

\begin{equation*}DC\end{equation*} = *direct cost*

\begin{equation*}P\end{equation*} = *probability of cancellation*

*Equation 2*

\begin{equation*}DC = \sum_{i=1}^bn_i\left(M_i+t_i ·\ p\right)\end{equation*}

Legend: \begin{equation*}M_i\end{equation*} = *material cost*

\begin{equation*}t_i\end{equation*} = *preparation time*

\begin{equation*}p\end{equation*} = *labor price*

The EW equation can be applied using the cancellation probability (P), calculated based on the history of the cancellation rate by surgery type. This application is especially suggested in situations in which a goal is proposed to be achieved.

The EW equation was applied for 2021. Thus, the financial losses related to processing consigned surgical instruments related to hip arthroplasty surgery were R$453.84 (US$84.04) per procedure; the financial losses related to spinal arthrodesis surgery were R$300.02 (US$55.56); and those related to knee arthroplasty surgery were equal to R$447.04 (US$82.78).

The EW equation demonstrates the waste in processing boxes of consigned surgical instruments. It is emphasized that the proposed equation enables inserting various hospital medical articles processed by the MSC, as well as being able to include size and weight characteristics such as single items, boxes by size (from small to large, for example) through the term (i) of the equation and its quantity by the term (n), allowing the EW to be calculated for any surgery and according to its peculiarities.

## Discussion

The present study revealed that 1,640 boxes of consigned surgical instruments were processed and not used due to surgical cancellation from 2019 to 2021, which was mostly linked to institutional problems.

Considering only hip arthroplasty, spine arthrodesis and knee arthroplasty surgeries, the waste observed in the period from 2019 to 2021 totaled R$34,340.18 (US$6,359.30). It should be noted that the value only refers to the investment wasted on consigned materials which were prepared and not used, without considering the other costs involved with surgical cancellation.

In recent decades, elective orthopedic surgery has become one of the specializations with long waiting lists of patients awaiting surgical treatment. Among the reasons linked to this reality are the increase in surgical demand associated with population aging and also the advancement of available techniques related to surgical technical-scientific improvement ^(^ 16 ^-^ 17 ^)^. 

Therefore, identifying and reducing the causes which lead to cancelling surgical procedures can contribute to improve several indicators and guide new practices for organizing the service, as they range from identifying previous clinical changes, to pre-operative visits, outpatient or other means, such as telehealth, and to optimizing the availability of a multidisciplinary team, more appropriate management of operating room usage time and daily surgical production, according to the hospital structure ^(^ 17 ^-^ 18 ^)^. 

Canceling a surgery certainly generates waste, increases the institution’s operational and financial costs and reduces the efficiency of the service, as well as generating physical, emotional and socioeconomic losses for the patient and their families ^(^ 17 ^,^ 19 ^-^ 20 ^)^. Furthermore, the financial investment made in surgeries that were canceled could be reversed into improvements to institutional processes, materials or care equipment, and also to the health team through educational actions. Therefore, there is a need to understand the costs of procedures at all levels of care, which drives a growing area of study for professionals who work directly or indirectly in healthcare ^(^ 21 ^-^ 23 ^)^. 

Previous investigations have also assessed the amounts invested in different aspects of perioperative care. A study carried out in Iran which analyzed the cancellation of 274 surgeries found that the total cost of surgical cancellations was US$92,049.00, and the costs related to resources and supplies (medicines, consumption and reprocessed material) totaled US$32,363.00, resulting in an average cost of $118.00 for each patient ^(^ 24 ^)^. 

Regarding the processing cost, a study carried out in Brazil analyzed the processing of an average of 20 boxes of orthopedic surgical instruments consigned for hip arthroplasty procedure, and observed the cost of R$347.69 per prepared surgery ^(^ 25 ^)^. This value is similar to that observed in the present study which found an estimated cost of R$453.80, even considering a lower average number of boxes (12 units) for the same type of procedure. Also similar to the present study, another Brazilian analysis using the absorption costing method for MSC activities identified an average unit cost of R$5.33 (US$2.85) for any product processed by the MSC, with sterilization costs/article of R$6.05 (US$3.23) ^(^ 26 ^)^. 

Knowing and analyzing costs facilitates actions to reduce waste and improve results. In this sense, an American study observed that only 20.5% of the instruments contained in the boxes intended for elective gynecological surgeries were actually used, thus correlating with US$232.16 in waste on sterilizing unused instruments ^(^ 27 ^)^. 

Optimizing surgical trays to improve operating room efficiency and reduce instrument processing costs is an underappreciated strategy for cost containment. Therefore, investigations aimed to reduce the number of instruments in surgical trays seeking to ensure that more than 50% of the instruments contained there were used. Researchers from the University of Alabama (USA) proved that after an intervention in the urology specialization there were savings of between US$7.48 and US$70.18 per procedure ^(^ 28 ^)^; likewise, savings of between US$55 and US$96 per thoracoscopy and thoracotomy surgeries were observed ^(^ 29 ^)^. 

Another investigation carried out by researchers from Philadelphia demonstrated that only 45.5% of the consigned instruments processed and opened on the operating table were effectively used in orthopedic surgery for total knee arthroplasty, and after removing the surplus the average preparation time decreased from 27.9 to 18.6 minutes, while 45 to 150 minutes were saved during processing. The average annual savings was US$281,298.05 ^(^ 30 ^)^. 

When measuring the cost of processing consigned surgical instruments in order to portray the waste generated by surgical cancellation, it was found that the inspection and preparation stages consumed the greatest amount of labor time, meaning the work which was wasted in the studied institution. Waste is endemic in healthcare services and should not be viewed solely from a financial perspective, as other precious resources in addition to money such as time and workforce satisfaction are being unnecessarily depleted by waste in processes every day ^(^ 31 ^)^. 

Regardless of how they are organized, healthcare systems in all countries must strive to maximize the benefits generated for patients linked to the investment made. It is important to reflect that the cost of not doing this can be measured in money, but also in avoidable deaths, pain and disability ^(^ 32 ^)^. 

Therefore, in order to engage in this purpose, it is important to make the negative impact of waste clear to employees, patients and companions, in addition to highlighting the investment potential of this economy to successfully disseminate innovative models to improve health and care, making it possible to demonstrate that waste was avoided by maintaining the right care, in the right place and at the right time ^(^ 31 ^)^. 

It can be noted that knowledge in a certain area is not enough to approach waste, and it is necessary to seek other skills and applied science to face multifaceted problems ^(^ 33 ^)^. Thus, the equation to calculate the EW occurring when canceling surgeries was proposed herein. The mathematical equation, as well as the trajectory for costs developed by the present study was outlined seeking easy operationalization by being applicable in the practice of nurses managing the perioperative period, equipping these professionals for planning based on institutional, care and financial data, aiming at quality, safety and better use of resources, thereby better controlling waste. 

The proposed equation can be applied to other specializations, meaning to any materials processed by the MSC, and will enable new studies which can add other costs related to surgical cancellation, such as: idle operating room occupancy rate, hospitalization days and other costs that are private to each healthcare organization.

To this end, it is essential that nurses appropriate data which equips them to make decisions about allocating financial and managerial resources, since it is not possible to provide highly complex care, bring together students and residents in care practices and develop research without funding, adequate contribution of resources and qualified management ^(^ 34 ^)^. When evidence of the impact of waste is well presented, it is a strong motivator for change. 

Nurses have an important role in identifying and reducing waste and influencing other professionals in the healthcare environment to increase their efficiency and productivity, as it is not enough to just gather concepts related to cost accounting, but to have in-depth knowledge of operations ^(^ 35 ^)^. Ultimately, there is no “silver bullet”, just incremental changes based on quality data ^(^ 32 ^)^. 

This study enables advancing the knowledge produced by the area by offering nurses a practical way of calculating waste. Furthermore, the proposed formula allows adjustments, adapting to the realities of different health institutions in Brazil and around the world.

However, this study presents some limitations, as it was not possible to evaluate and financially estimate the environmental impact caused by waste discarded in the environment which did not fulfill its purpose, as well as finite natural resources. Future studies may estimate the overall costs of canceled surgical procedures using the proposed EW equation and including new assessment items, such as the idle operating room rate and daily hospitalization rates.

## Conclusion

This study identified 1,640 boxes of consigned surgical instruments which were processed and unused in the periods of 2019, 2020 and 2021. The greatest demand occurred in hip arthroplasty, spine arthrodesis and knee arthroplasty surgeries with 52% (854) of wasted boxes.

Causes related to the institution (40; 47.0%) stood out as being responsible for surgical cancellation, which mainly involved problems with the material, especially regarding the preparation and offer of HPs by the MSC; problems related to the patient (39; 45.9%), especially clinical, and finally, problems related to the medical team (6; 7%).

It was observed that, on average, 12 boxes of surgical instruments were prepared for hip surgery, seven for spine surgery and 11 for knee surgery, with Stage 4 (inspection and preparation) responsible for consuming the majority of the CME professional’s time in preparing consigned surgical instruments.

The MDC of processing per box of surgical instruments consigned for hip surgery was R$31.72 (US$5.87); for spine surgery it was equal to R$36.76 (US$6.81); and for knee surgery it was R$34.54 (US$6.40). Thus, considering only the cancellation of hip, spine and knee orthopedic procedures from 2019 to 2021, the EW equation revealed the financial waste associated with processing boxes of consigned surgical instruments from hip surgeries was R$11,610.74 (US$2,150.14); R$12,343.68 (US$2,285.89) for spine surgeries; and R$10,525.76 (US$1,949.21) for knee surgery, totaling R$34,340.18 (US$6,359.30).

The proposed equation enables inserting various hospital medical articles processed by the MSC, as well as being able to include size and weight characteristics such as single items, boxes by size (from small to large, for example), through the term (i) of the equation and its quantity by the term (n), allowing the EW to be calculated for any surgery and according to its peculiarities.

Thus, nurses can contribute to cost management by conducting studies which provide elements for rationalizing the resource allocation process, for balancing costs and finances and for increasing results, in turn directing a redefinition of priorities and productivity monitoring.
